# Efficacy of high-intensity interval training for improving mental health and health-related quality of life in women with polycystic ovary syndrome

**DOI:** 10.1038/s41598-023-29503-1

**Published:** 2023-02-21

**Authors:** Rhiannon K. Patten, Luke C. McIlvenna, Alba Moreno-Asso, Danielle Hiam, Nigel K. Stepto, Simon Rosenbaum, Alexandra G. Parker

**Affiliations:** 1 Institute for Health and Sport (iHeS), Victoria University, Melbourne, VIC Australia; 2 Epigenetics & Cellular Senescence Group, Blizard Institute, Barts and the London School of Medicine and Dentistry, Queen Mary University of London, London, UK; 3 Australian Institute for Musculoskeletal Science (AIMSS), Victoria University, University of Melbourne and Western Health, Melbourne, VIC Australia; 4 Institute for Physical Activity and Nutrition (IPAN), School of Exercise and Nutrition Sciences, Deakin University, Geelong, VIC Australia; 5 Discipline of Psychiatry and Mental Health, University of New South Wales Sydney, Sydney, NSW Australia; 6 School of Health Sciences, University of New South Wales, Sydney, NSW Australia; 7 Victoria University, Ballarat Rd, Footscray, VIC 3011 Australia

**Keywords:** Human behaviour, Psychology, Endocrinology

## Abstract

Women with PCOS have substantially greater symptoms of depression and anxiety, and a lower health-related quality of life (HRQoL) compared to women without PCOS. The aim of this study was to determine if high-intensity interval training (HIIT) could provide greater improvements in mental health outcomes than standard moderate-intensity continuous training (MICT). Twenty-nine overweight women with PCOS aged 18–45 years were randomly assigned to 12 weeks of either MICT (60–75% HR_peak_, N = 15) or HIIT (> 90% HR_peak_, N = 14). Outcome measures included symptoms of depression, anxiety and stress (DASS-21), general HRQoL (SF-36) and PCOS specific HRQoL (PCOSQ) collected at baseline and post-intervention. Reductions in depression (Δ − 1.7, P = 0.005), anxiety (Δ − 3.4, P < 0.001) and stress (Δ − 2.4, P = 0.003) scores were observed in the HIIT group, while MICT only resulted in a reduction in stress scores (Δ − 2.9, P = 0.001). Reductions in anxiety scores were significantly higher in the HIIT group compared to the MICT group (β = − 2.24, P = 0.020). Both HIIT and MICT significantly improved multiple domain scores from the SF-36 and PCOSQ. This study highlights the potential of HIIT for improving mental health and HRQoL in overweight women with PCOS. HIIT may be a viable strategy to reduce symptoms of depression and anxiety in women with PCOS, however, large-scale studies are required to confirm these findings.

**Trial registration number**: ACTRN12615000242527.

## Introduction

Polycystic ovary syndrome (PCOS) is the most common endocrine condition, affecting 8–13% of reproductive aged women^1,2^. PCOS is characterised by hyperandrogenism, menstrual irregularities and polycystic ovary morphology, with a combination of at least two of the three required for diagnosis^3^. PCOS is underpinned by reproductive and metabolic abnormalities, resulting in an increased risk of comorbidities including obesity, insulin resistance, type 2 diabetes and infertility^4–6^. PCOS is also recognised to be associated with diminished mental health and health-related quality of life (HRQoL)^7,8^. Women with PCOS are three times more likely to experience moderate to severe symptoms of depression and five times more likely to experience severe symptoms of anxiety compared to women without PCOS^9^. Obesity is also highly prevalent in women with PCOS, and is known to be associated with depression in both healthy women^10,11^ and in women with PCOS^12,13^, however, the exact mechanisms behind this association is unclear. Evidence suggests that there are biological, behavioural and psychological links^14^, with being female and poor physical health being considered risk factors for comorbid depression and obesity^15,16^. It is postulated that the symptoms and comorbidities of PCOS may contribute to poorer mental health and reduced HRQoL^17,18^.

Exercise is recommended for women with PCOS to improve general health, HRQoL and to maintain or achieve a healthy weight^19^. Exercise participation has been associated with lower scores of depression in both women with and without PCOS^20^. In line with the Australian physical activity guidelines, the International evidence-based guidelines for the assessment and management of PCOS recommend a minimum of 150 min per week of moderate intensity physical activity or 75 min per week or vigorous intensity exercise^19^. Exercise is well established as a therapeutic tool to improve metabolic and reproductive health outcomes in women with PCOS^21,22^, however, the impact on mental health and HRQoL is less clear^23,24^. In populations with other chronic conditions, exercise is known to be an effective tool for promoting positive mental health^25,26^. Existing research into the effect of exercise on mental health and HRQoL in women with PCOS suggests that exercise interventions may promote improvements in these outcomes^23,27,28^, however, large heterogeneity between studies makes it difficult to draw conclusions regarding the exercise requirements to promote mental health benefits.

Regular exercise is associated with improving specific mental health symptoms including depression^29^ and anxiety^30^. This relationship has also been observed in women with PCOS^31^. A minimum of 20 min of exercise per week has been reported to reduce the odds of psychological distress in both males and females without PCOS^32^, with a greater volume or intensity of exercise associated with an even greater risk reduction^32^. High-intensity interval training (HIIT) has received considerable attention over the past two decades due to its time efficiency and potent training stimulus^33^. HIIT involves alternating short bouts of high-intensity exercise with periods of rest or light exercise^34^. HIIT has been found to result in greater improvements in cardio-metabolic health outcomes in comparison to moderate-intensity continuous training (MICT) among healthy populations, and in those with chronic conditions^33,34^. In women with PCOS, HIIT has also been found to improve cardio-metabolic and reproductive health outcomes, resulting in a reduction in the severity of PCOS symptoms^35–37^. Despite the growing body of evidence supporting the physiological benefits of HIIT, there is little information regarding its effect on mental health and HRQoL. Limited evidence suggests that HIIT can improve HRQoL and symptoms of anxiety and depression among the general population and those with chronic conditions^34,38,39^. It could be postulated that HIIT may provide greater improvements in mental health and HRQoL in women with PCOS compared to MICT due to its large impact on reducing the severity of symptoms associated with PCOS. Therefore, the aim of this study was to determine the efficacy of HIIT in comparison to MICT for improving mental health and HRQoL in women with PCOS. The outcomes included change in depression, anxiety, stress and HRQoL scores.

## Methods

### Study design

This study is a secondary analysis of a two-arm randomised clinical trial that was conducted at Victoria University in Melbourne, Australia, from June 2016 to October 2019^37^. The study was approved by the Victoria University Human Research Ethics Committee (15–298), which complied with the statement of ethical principles for medical research outlined in the Declaration of Helsinki. The trial was prospectively registered with the Australian New Zealand Clinical Trials Registry (ACTRN12615000242527). All women provided written informed consent prior to participation.

### Participants

Women were recruited through community and social media advertisements. Inclusion criteria were women aged 18–45 (pre-menopausal), with a BMI between 25 and 50 kg/m^2^, insufficiently active for a minimum of 6 months prior to the intervention (did not meet the minimum physical activity recommendations of 150 min/week of moderate intensity or 75 min/week of vigorous intensity exercise)^40^, and with diagnosed PCOS. PCOS was diagnosed according to the Rotterdam Criteria^3^, and confirmed by an endocrinologist. The Rotterdam criteria required confirmation of two of the following: (i) oligo- or anovulation; (ii) clinical (hirsutism and/or biochemical hyperandrogenism; (iii) polycystic ovaries on ultrasound and the exclusion of other causes of hyperandrogenism. Exclusion criteria included diabetes, pregnancy, smoking, illness or injury that prevented or limited exercise performance and existing participation in regular physical activity. Those taking anti-hypertensive, insulin sensitising or hormonal contraceptive medications were excluded. Anti-depressants, anti-anxiety or similar medications were not an exclusion criteria for this study. Participants taking these medications for a minimum of three months prior to beginning the study were included and asked to maintain their usual dose throughout the intervention.

### Study protocol

Following the completion of baseline testing, participants were randomised to one of two interventions: 12-week HIIT or MICT intervention. Randomisation was completed by an independent biostatistician by a simple randomisation procedure using computerised sequence generation at an allocation ratio of 1:1. To ensure equal proportions of body mass index (BMI) in each arm, randomisation was stratified according to BMI brackets (< 35 kg/m^2^ or > 35 kg/m^2^). Participants were asked to maintain their usual diet throughout the duration of the intervention and 3-day food diaries were used to capture this information.

### Mental health and health-related quality of life measures

The Depression Anxiety Stress Scales (DASS-21) is a self-report measure which is well-established, reliable and valid instrument for measuring depression, anxiety and stress^41^. The questionnaire consists of 21 questions, with 7 items per domain. The severity of symptoms is scored on a scale 4-point Likert scale. The total score for each category is added together. Higher scores denote more severe symptoms.

Health-related quality of life was assessed using the generic SF-36 (36-item Short-Form Health Survey). This measure is well-validated, including in women with PCOS^42^. It consists of 8 health domains: physical functioning (10 questions), role limitations due to physical health problems (4 questions), role limitations due to emotional problems (3 questions), emotional well-being (5 questions), social functioning (2 questions), bodily pain (2 questions), energy/fatigue (4 questions), and general health perceptions (5 questions). Scoring was calculated according to the RAND procedure^43^ where each item is scored between 0 and 100. Scores are then averaged for each of the 8 domains. A higher score indicates a more favourable health state.

Health-related quality of life associated with PCOS symptom distress was measured using a validated self-administered questionnaire (PCOSQ)^44,45^. The PCOSQ consists of 26 questions with 5 domains each relating to a common symptom of PCOS; emotions (7 questions), menstrual problems (4 questions), infertility (5 questions), weight (5 questions) and body hair (5 questions). Questions are scored on a 7-point Likert scale in which 1 represents poorest function and 7 represents optimal function. For each domain, scores are added together and then divided by the number of questions to reach the domain score. Lower scores indicate greater PCOS symptom distress. A change of 0.5 is considered clinically relevant^44^.

### Anthropometric measurements

Participants were weighed without shoes and lightly clothed (Proscale Inductive Series I, Accurate Technology Inc. USA). Height was measured without shoes using a calibrated stadiometer (Proscale Inductive Series I, Accurate Technology Inc., USA). Body mass index (BMI) was calculated as weight[kg]/height squared[m]. Additional body composition measures were collected and reported in Patten et al.^37^.

### Aerobic capacity

Aerobic capacity was assessed at baseline and following the 12 week intervention using an incremental maximal graded exercise test conducted on an electronically-braked cycle ergometer (Lode Excalibur v2.0, The Netherlands) to measure peak oxygen uptake (VO_2peak_). The initial three stages of the test consisted of three 3 min stages of cycling at 25, 50 and 75 watts, respectively, followed by increases of 25 watts each minute thereafter until volitional exhaustion. Breath-by-breath expired respiratory gases were collected and analysed (Quark Cardio-Pulmonary Exercise Testing, Cosmed, Italy) and relative VO_2peak_ was calculated as previously described^37^. Peak heart rate (HR_peak_) was also recorded (Polar H10, Polar Electro OY, Kempele, Finland) in order to determine exercise training intensities. The initial exercise test included electrocardiogram (ECG) to screen for cardiovascular risk.

### Insulin sensitivity

Insulin sensitivity was assessed using the euglycaemic-hyperinsulinaemic clamp described in Patten et al.^37^. In brief, a bolus of insulin of 9 mU/kg was infused, followed by a constant insulin infusion rate of 40 mU/min/m^2^ with glucose (20%) infused at a variable rate to meet the target blood glucose of 5 mmol/L. The euglycaemic-hyperinsulinaemic clamp was run for at least 120 min or until steady state of 5 mmol/L was achieved. Insulin sensitivity index (ISI) was calculated using the following formula: (glucose infusion rate (mg/lean body mass [kg]/min)/steady state insulin) × 100.

### Exercise interventions

Interventions were designed to match the minimum exercise recommendations for the moderate and vigorous exercise according to the International evidence-based guidelines for the assessment and management of PCOS^19^ and were matched for training volume (metabolic equivalent task [MET] minutes/week)^46^. In brief, women in both groups progressed from 312 MET minutes per week in week 1, to 530 MET minutes per week by week 4 (Supplementary Fig. 1). Participants in both groups attended three weekly sessions for 12 weeks. All sessions were conducted on an electronically braked cycle ergometer (Excalibur, V2.0; Lode, Groningen, the Netherlands) under the supervision of an accredited exercise physiologist. For both interventions, sessions were preceded by a 5 min warm-up at a moderate intensity and ended with a 5 min cool down. Adherence to exercise training was calculated as the number of sessions attended divided by the total number of scheduled sessions, reported as a percentage. Heart rate monitors (Polar H10, Polar Electro OY, Kempele, Finland) were used in all sessions and target heart rates were achieved by altering the load on the bike according to individual fitness.

### HIIT intervention

The HIIT intervention included three sessions per week: two sessions of 12 × 1 min intervals at 90–100%HR_peak_(~ 10 METs), interspersed with 1 min of active recovery at a light load; and one session of 8 × 4 min intervals at 90–95%HR_peak_ (~ 8 METs), interspersed with a 2 min light load, active recovery. Recovery periods were light intensity, corresponding to 7–11 on a rating of perceived exertion (RPE) scale.

### MICT intervention

The MICT intervention consisted of three weekly sessions of 45 min of continuous moderate-intensity cycling at 60–75%HR_peak_.

### Statistical analysis

All data was analysed using R studio version 4.0.2 and conducted on an intention-to-treat basis, which included all randomly assigned participants. Linear mixed models were used to determine the effect of exercise intensity (group) over time and to determine the interaction between timepoint and group (between-group differences). To examine the within group changes, we calculated the estimated marginal means from the linear mixed models. The unique participant codes were used as a random effect to account for repeated measures. All models were adjusted for age. Simple linear regressions were used to determine the association between VO_2peak_, insulin sensitivity or body composition, and mental health or HRQoL outcomes using the pooled data from all participants. Data are presented as mean ± SD and median and percentiles for boxplots. P values were deemed statistically significant when < 0.05. The following packages were used in our analysis; *lme4*^47^, *lmerTest*^48^ and *tidyverse*^49^. The estimation of sample size was based on the primary outcome of VO_2peak_, based on this, we aimed to recruit 30 participants^37^. As this was a secondary analysis, power calculations were not done for the outcome measures reported in this study.

## Results

**Figure 1:**
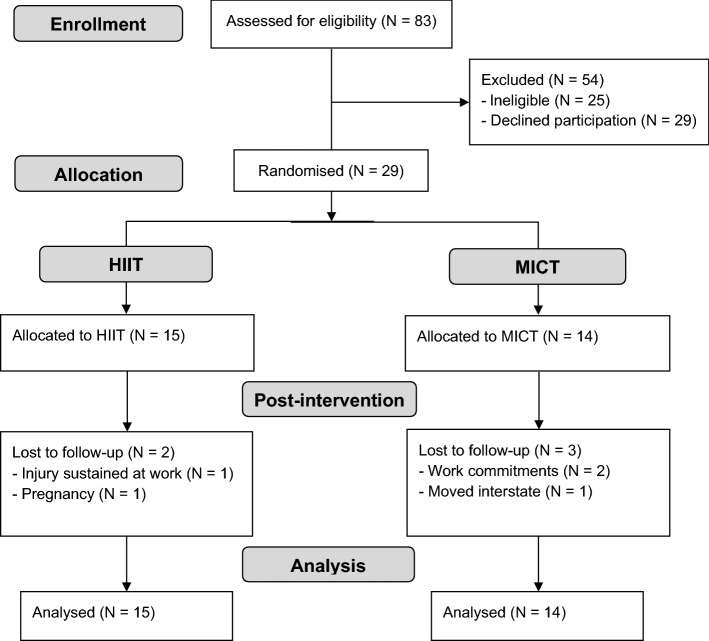
CONSORT trial flow diagram.

Of the 83 women that were assessed for eligibility, 29 women declined to participate and 25 were ineligible (with use of hormonal contraceptives being the most common reason for exclusion). Twenty-nine participants completed the baseline assessments (HIIT = 15, MICT = 14), of whom 24 completed the 12-week intervention (HIIT = 13, MICT = 11). Two participants withdrew during the MICT intervention due to changes in their work schedule and one withdrew due to moving interstate. One participant withdrew from the HIIT intervention due to an injury sustained at work and another became pregnant (Fig. 1). There were no significant differences in study outcomes at baseline between participants who dropped out and those that completed the study. Two participants in both the HIIT and MICT group were taking anti-depressants throughout the intervention. The inclusion of these participants did not significantly alter the results and they were therefore included in all analyses. A breakdown of PCOS phenotypes is presented in Supplementary Table 1. There were no significant differences in dietary intake from baseline to post-intervention in either group. Exercise intervention adherence was similar across the two groups with an average adherence of 93.9 ± 3.0% in the HIIT group and 92.0 ± 4.8% in the MICT group (P > 0.05). No adverse events occurred during the study period.

### Mental health outcomes

**Figure 2:**
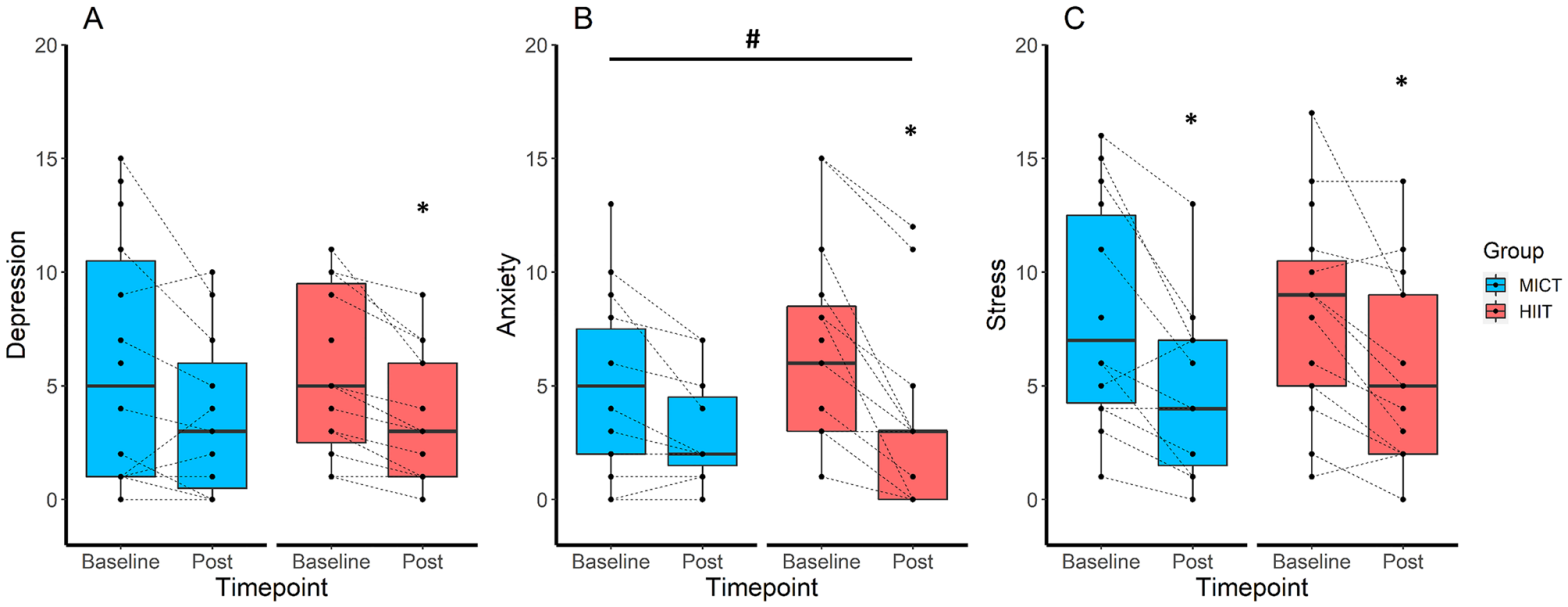
Effect of exercise intensity on depression (**A**), anxiety (**B**) and stress (**C**) scores. Baseline and post-intervention scores, stratified by group and using boxplots showing the median (central line) and 25th to 75th quartiles (box). *Indicates a significant within-group difference P < 0.05, and ^#^indicates a significant between-group difference P < 0.05.

The HIIT intervention reduced scores for depression (Δ − 1.7, P = 0.005), anxiety (Δ − 3.4, P < 0.001) and stress (Δ − 2.4, P = 0.003; Table 1), while the MICT intervention only reduced stress scores (Δ − 2.9, P = 0.001; Fig. 2). There was a significant interaction between the two groups for anxiety scores, whereby HIIT resulted in greater improvements compared to MICT (β = − 2.24, P = 0.020; Fig. 2), but not for depression or stress scores (Table 1).

**Table 1:** Mental health and health-related quality of life outcomes at baseline and post-intervention.

Outcome measure	HIIT			MICT			P (time × group)
	Baseline N = 15	Post N = 13	P	Baseline N = 14	Post N = 11	P	
Age	29.7 ± 4.8			32.5 ± 6.2			
Weight (kg)	97.4 ± 19.2	97.3 ± 19.1	0.593	102.4 ± 28.9	99.8 ± 28.0	0.291	0.257
BMI	35.5 ± 6.8	35.6 ± 7.0	0.632	38.4 ± 9.3	37.3 ± 9.8	0.256	0.248
VO_2peak_	24.8 ± 5.7	30.8 ± 6.7	**< 0.001**	22.3 ± 3.2	26.1 ± 4.1	**< 0.001**	**0.017**
ISI (µIU/mL)	108.7 ± 47.5	101.2 ± 29.2	0.226	120.1 ± 28.4	125.6 ± 31.1	0.475	0.202
WHR	0.8 ± 0.1	0.8 ± 0.1	0.841	0.9 ± 0.1	0.8 ± 0.1	**0.008**	0.059
Fat mass (kg)	43.6 ± 14.3	42.5 ± 12.8	0.249	49.3 ± 20.6	50.3 ± 18.9	0.322	0.135
Lean mass (kg)	46.3 ± 4.1	47.0 ± 4.1	0.110	46.1 ± 7.2	46.8 ± 6.8	0.112	0.997
DASS-21							
Depression score	5.5 ± 3.6	3.5 ± 2.9	**0.005**	6.1 ± 5.4	3.7 ± 3.6	0.070	0.479
Anxiety score	6.5 ± 4.6	3.2 ± 4.1	**< 0.001**	5.0 ± 3.8	3.0 ± 2.4	0.098	**0.020**
Stress score	8.2 ± 4.5	5.7 ± 4.2	**0.003**	8.1 ± 4.8	4.8 ± 3.9	**0.001**	0.641
PCOSQ							
Emotions	4.0 ± 1.3	4.8 ± 1.0	**0.023**	4.0 ± 1.1	4.8 ± 0.8	**0.040**	0.972
Body hair	3.0 ± 1.3	3.5 ± 1.7	0.252	3.8 ± 1.7	4.6 ± 1.6	0.066	0.535
Weight	2.0 ± 1.0	2.7 ± 1.2	**< 0.001**	2.0 ± 1.0	2.5 ± 0.9	**0.042**	0.230
Infertility	4.7 ± 1.5	5.0 ± 1.8	0.088	4.5 ± 1.7	4.7 ± 1.9	0.261	0.725
Menstrual problems	3.8 ± 1.2	4.8 ± 1.2	**0.017**	3.6 ± 1.3	4.1 ± 1.3	0.154	0.511
SF-36							
Physical functioning	79.7 ± 19.2	88.5 ± 18.4	**0.022**	74.6 ± 20.3	85.9 ± 17.7	0.076	0.766
Role physical	86.7 ± 22.9	94.2 ± 15.0	0.299	87.5 ± 21.4	95.5 ± 10.1	0.296	0.951
Role emotional	51.1 ± 37.5	71.8 ± 32.9	**0.016**	59.5 ± 37.4	87.9 ± 22.5	**0.038**	0.882
Energy	39.6 ± 16.2	53.1 ± 17.7	**0.008**	38.9 ± 18.4	45.9 ± 19.5	0.333	0.230
Emotions	62.7 ± 12.9	67.4 ± 14.7	0.095	62.9 ± 18.7	69.1 ± 20.1	0.626	0.422
Social	72.5 ± 23.2	75.0 ± 24.5	0.678	67.0 ± 31.6	78.4 ± 30.2	0.330	0.658
Pain	71.3 ± 20.1	79.2 ± 21.2	0.074	73.2 ± 16.7	79.8 ± 12.2	0.304	0.623
General health	42.7 ± 17.6	54.6 ± 19.2	**< 0.001**	49.3 ± 21.1	61.8 ± 19.3	**0.044**	0.166

Significant values are in bold.

Data are mean ± SD.

*BMI* body mass index, *DASS-21* depression, anxiety and stress scale—21 items, *HIIT* high-intensity interval training, *ISI* insulin sensitivity index, *MICT* moderate-intensity continuous training, *PCOSQ* polycystic ovary syndrome questionnaire, *SF-36* short-form survey—36 items, *VO*_*2peak*_ peak oxygen consumption, *WHR* waist to hip ratio.

### PCOS specific HRQoL

The HIIT intervention improved scores in 3 of the 5 domains of the PCOSQ; emotions (Δ 0.8, P = 0.023), weight (Δ 0.6, P < 0.001) and menstrual problems (Δ 0.9, P = 0.017; Table 1). The MICT intervention resulted in an improvement in the emotions (Δ 0.8, P = 0.040) and weight (Δ 0.5, P = 0.042) domains. There were no significant group by time interactions between the HIIT and MICT groups for any domain (Table 1).

### General HRQoL

The HIIT intervention improved physical functioning (Δ 8.8, P = 0.022), role emotional (Δ 20.7, P = 0.016), energy (Δ 13.4, P = 0.008) and general health (Δ 11.9, P < 0.001) domains of the SF-36, while the MICT group improved role emotional (Δ 28.4, P = 0.038) and general health (Δ 12.5, P = 0.044) domain scores (Table 1). There were no significant between group differences for any domains of the SF-36 (Table 1).

### Associations between physiological and psychological outcomes

At baseline, there were significant inverse associations between VO_2peak_ and depression (R^2^ = 0.15, P = 0.005), anxiety (R^2^ = 0.19, P = 0.001) and stress (R^2^ = 0.10, P = 0.022). BMI was associated with depression (R^2^ = 0.12, P = 0.011) and anxiety (R^2^ = 0.12, P = 0.011), but not stress (P = 0.054), and insulin sensitivity was not associated with depression, anxiety or stress (P > 0.05). Regardless of the exercise intervention, there were no significant associations between improvements in VO_2peak_ and changes in depression, anxiety or stress scores (P > 0.05). Nor were there significant associations between changes in BMI, weight, WHR or fat mass and depression, stress or anxiety. Interestingly, changes in lean mass was associated with a change in depression (R^2^ = 0.23, P = 0.025), but not stress or anxiety (P > 0.05). When analysing the groups separately, this association remained significant for the MICT group (R^2^ = 0.40, P = 0.036), but not the HIIT group (R^2^ = 0.16, P = 0.224). There were no significant associations between changes in body composition and changes in the weight, emotion or menstrual problem domains of the PCOSQ.

## Discussion

This study demonstrates that participation in a supervised 12-week HIIT intervention leads to a reduction in depression, anxiety and stress scores in overweight women with PCOS, with MICT also significantly improving stress scores. Both HIIT and MICT resulted in improvements in multiple domains of both general and PCOS specific HRQoL.

It is well-established that women with PCOS display a higher prevalence of anxiety and depression disorders compared to women without PCOS^7^. Although exercise is an effective means for managing mental health concerns in a general population^50^, this relationship has been relatively understudied in women with PCOS. Existing research reports a combination of positive and null findings, with approximately half of existing studies reporting significant improvements in symptoms of anxiety and/or depression^23,24^. To the best of our knowledge, this is the first study to compare work-matched HIIT and MICT exercise interventions on mental health and HRQoL in women with PCOS. We report that significant improvements in depression, anxiety and stress scores as a result of HIIT, while in response to MICT significant improvements were only observed in stress scores. These findings are in contrast to a recent 16-week time-matched training intervention where participants from both the continuous and intermittent aerobic training groups reported improvements in depression and anxiety^51^. The difference in our findings could be due to the fact that their intervention was 4 weeks longer in duration or the higher intensity performed in their continuous group compared to our MICT group (65–80%HR_max_ vs 60–75% HR_peak_). Other successful interventions for improving symptoms of depression and anxiety have used multi-component interventions that have included either diet^52^, medication^53^ or cognitive behavioural therapy^54^ in addition to exercise. The large variation in the interventions implemented in prior studies makes it difficult to adequately assess the impact of exercise intensity alone on symptoms of depression and anxiety, however, our current findings suggest that HIIT may be a promising therapy for improving mental health in women with PCOS. Further research with larger sample sizes and long-term follow-ups are required to determine the optimal intensity, frequency, type and duration of exercise.

Exercise intensity can elicit different neural responses and may influence the effect on depressive symptoms, however, the exact mechanisms are unclear^55^. Furthermore, it has been suggested that exercise at an individual’s preferred intensity results in greater improvements in mental health outcomes and attrition rates. A study conducted in women with depression reported significantly lower depression scores after 12 sessions of exercise at their preferred intensity compared to prescribed intensity^56^. We previously reported significantly greater enjoyment and affect in the HIIT group compared to the MICT group which may have contributed to the significant reduction in depression observed here^57^. Improvements in fitness have been associated with reductions in symptom severity in individuals with depression^58^, and therefore, it could be hypothesised that the greater improvements in depression scores may have been due to the larger improvements in fitness observed in the HIIT group. However, our findings did not support this hypothesis, and we did not observe an association between improvements in fitness and changes in mental health and HRQoL outcomes. In the general population, metabolic risk factors, primarily obesity, are known to be correlated with depression, with weight loss being associated with reduced depression score^59^. Our intervention did not significantly change weight, and we did not report an association between changes in depression scores and weight. Interestingly, in women with PCOS, insulin resistance has previously been associated with a 2.3-fold increased odds of depression^60^, however, we did not observe this association at baseline, or using the delta changes. We acknowledge that our small sample size may have led to the lack of physiological associations.

Women with PCOS display reductions in HRQoL compared to women without PCOS^42^. Unique challenges with poor body image and dissatisfaction, low self-esteem, fertility, acne, hirsutism and long-term health concerns have been shown to compromise HRQoL in women with PCOS^61,62^. In response to HIIT, we report significant improvements in the emotions and menstrual problems domains of the PCOSQ and significant improvement in the weight domain as a result of both interventions. This is a surprising result as neither group resulted in significant improvements in body weight or BMI, nor were there significant correlations between changes in body composition and changes in weight, emotions or menstrual problem domains. Similar results have been reported by previous studies that utilised a lifestyle modification intervention which also included diet^63,64^, however, the majority of studies that reported change in the weight domain of the PCOSQ also reported significant weight loss^52,53,65^. Weight loss has also been assumed to be a significant contributor to improvements in distress caused by PCOS symptoms. Our findings indicate that exercise, in the absence of weight loss, can improve this distress. We have previously shown that HIIT results in greater improvements in aerobic capacity, insulin sensitivity, menstrual regulation and hormonal profiles compared to MICT^37^ and these improvements may play a role in the reduced distress observed. Thus, given the high rates of mental health disorders, the distress caused by symptoms and the high-risk of developing cardio-metabolic conditions amongst women with PCOS, our results suggest that HIIT may be a considered as an effective intervention for improving both physiological and psychological outcomes in these women. Therefore, HIIT holds promise as a time effective and efficacious intervention for both physiological and psychological outcomes.

Strengths of this study include the randomised design and the work-matched interventions. We would like to acknowledge there are some limitations to this study. As this study is a secondary analysis, it was not designed to be powered to detect significant changes between groups in mental health and HRQoL outcomes. Future exercise interventions comparing exercise intensities that have been designed to detect changes in mental health outcomes are required to confirm these findings. It could also be considered a limitation that we did not include a non-exercise control group, however, we would like to highlight the numerous robust studies that have shown that exercise, compared to an inactive control group, improves the symptoms of PCOS, and therefore the aim of this study was to compare the two exercise intensities. We recommend that future studies also include a follow-up period to determine whether HIIT is sustainable post-intervention and to determine whether the improvements observed here are maintained long-term. Lastly, as many women were ineligible due to use of hormonal contraceptives, it is important that future studies examine the impact of HIIT in all women with PCOS regardless of medication use.

In conclusion, we found improvements in depression, anxiety and stress scores after HIIT, and reduced stress scores after MICT. Both exercise interventions resulted in improvements in multiple domain scores of HRQoL suggesting that exercise intensity may be less important for improving HRQoL. Although evidence from this study highlights the potential of HIIT for improving symptoms of mental health, further investigation into the benefits of HIIT compared to MICT for improving mental health and HRQoL outcomes in women with PCOS is warranted.

## Supplementary Information

Supplementary Information.

## Data Availability

The datasets generated during and/or analysed during the current study are available from the corresponding author on reasonable request.

## References

[1] March WA (2010). The prevalence of polycystic ovary syndrome in a community sample assessed under contrasting diagnostic criteria. Hum. Reprod..

[2] Azziz R (2006). Positions statement: Criteria for defining polycystic ovary syndrome as a predominantly hyperandrogenic syndrome: An Androgen Excess Society guideline. J. Clin. Endocrinol. Metab..

[3] Rotterdam EA-SPCWG (2004). Revised 2003 consensus on diagnostic criteria and long-term health risks related to polycystic ovary syndrome. Fertil. Steril..

[4] Stepto NK (2013). Women with polycystic ovary syndrome have intrinsic insulin resistance on euglycaemic–hyperinsulaemic clamp. Hum. Reprod..

[5] Moran LJ, Misso ML, Wild RA, Norman RJ (2010). Impaired glucose tolerance, type 2 diabetes and metabolic syndrome in polycystic ovary syndrome: A systematic review and meta-analysis. Hum. Reprod. Update.

[6] Joham AE, Teede HJ, Ranasinha S, Zoungas S, Boyle J (2015). Prevalence of infertility and use of fertility treatment in women with polycystic ovary syndrome: Data from a large community-based cohort study. J. Womens Health.

[7] Barry JA, Kuczmierczyk AR, Hardiman PJ (2011). Anxiety and depression in polycystic ovary syndrome: A systematic review and meta-analysis. Hum. Reprod..

[8] Barnard L (2007). Quality of life and psychological well being in polycystic ovary syndrome. Hum. Reprod..

[9] Cooney LG, Lee I, Sammel MD, Dokras A (2017). High prevalence of moderate and severe depressive and anxiety symptoms in polycystic ovary syndrome: A systematic review and meta-analysis. Hum. Reprod..

[10] Luppino FS (2010). Overweight, obesity, and depression: A systematic review and meta-analysis of longitudinal studies. Arch. Gen. Psychiatry.

[11] Stunkard AJ, Faith MS, Allison KC (2003). Depression and obesity. Biol. Psychiat..

[12] Rasgon NL (2003). Depression in women with polycystic ovary syndrome: Clinical and biochemical correlates. J. Affect. Disord..

[13] Greenwood EA, Pasch LA, Shinkai K, Cedars MI, Huddleston HG (2019). Clinical course of depression symptoms and predictors of enduring depression risk in women with polycystic ovary syndrome: Results of a longitudinal study. Fertil. Steril..

[14] Milaneschi Y, Simmons WK, van Rossum EFC, Penninx BWJH (2019). Depression and obesity: Evidence of shared biological mechanisms. Mol. Psychiatry.

[15] Kuehner C (2017). Why is depression more common among women than among men?. Lancet Psychiatry.

[16] Ma J, Xiao L (2010). Obesity and depression in US women: Results from the 2005–2006 national health and nutritional examination survey. Obesity.

[17] Deeks AA, Gibson-Helm ME, Paul E, Teede HJ (2011). Is having polycystic ovary syndrome a predictor of poor psychological function including anxiety and depression?. Hum. Reprod..

[18] Teede H, Deeks A, Moran L (2010). Polycystic ovary syndrome: A complex condition with psychological, reproductive and metabolic manifestations that impacts on health across the lifespan. BMC Med..

[19] Teede HJ (2018). Recommendations from the international evidence-based guideline for the assessment and management of polycystic ovary syndrome. Fertil. Steril..

[20] Banting LK, Gibson-Helm M, Polman R, Teede HJ, Stepto NK (2014). Physical activity and mental health in women with polycystic ovary syndrome. BMC Womens Health.

[21] Patten, R. K.* et al.* Exercise interventions in polycystic ovary syndrome: A systematic review and meta-analysis. *Front. Physiol.* **11** (2020). 10.3389/fphys.2020.00606 PMC7358428 32733258

[22] Benham, J. L., Yamamoto, J. M., Friedenreich, C. M., Rabi, D. M. & Sigal, R. J. Role of exercise training in polycystic ovary syndrome: A systematic review and meta-analysis. *Clin. Obes.* (2018). 10.1111/cob.12258 29896935

[23] Conte F, Banting L, Teede HJ, Stepto NK (2015). Mental health and physical activity in women with polycystic ovary syndrome: A brief review. Sports Med..

[24] Patten RK (2021). Effectiveness of exercise interventions on mental health and health-related quality of life in women with polycystic ovary syndrome: A systematic review. BMC Public Health.

[25] Rebar AL (2015). A meta-meta-analysis of the effect of physical activity on depression and anxiety in non-clinical adult populations. Health Psychol. Rev..

[26] Ashdown-Franks G (2020). Exercise as medicine for mental and substance use disorders: A meta-review of the benefits for neuropsychiatric and cognitive outcomes. Sports Med..

[27] Ribeiro, V. B.* et al.* Continuous versus intermittent aerobic exercise in the improvement of quality of life for women with polycystic ovary syndrome: A randomized controlled trial. *J. Health Psychol.* (2019). 10.1177/1359105319869806 31495231

[28] Lara LA (2015). Impact of physical resistance training on the sexual function of women with polycystic ovary syndrome. J. Sex. Med..

[29] Teychenne M, Ball K, Salmon J (2008). Physical activity and likelihood of depression in adults: A review. Prev. Med..

[30] McDowell CP, Dishman RK, Gordon BR, Herring MP (2019). Physical activity and anxiety: A systematic review and meta-analysis of prospective cohort studies. Am. J. Prev. Med..

[31] Lamb JD (2011). Physical activity in women with polycystic ovary syndrome: Prevalence, predictors, and positive health associations. Am. J. Obstet. Gynecol..

[32] Hamer M, Stamatakis E, Steptoe A (2009). Dose-response relationship between physical activity and mental health: The Scottish Health Survey. Br. J. Sports Med..

[33] Wisløff U (2007). Superior cardiovascular effect of aerobic interval training versus moderate continuous training in heart failure patients: A randomized study. Circulation.

[34] Weston KS, Wisløff U, Coombes JS (2014). High-intensity interval training in patients with lifestyle-induced cardiometabolic disease: A systematic review and meta-analysis. Br. J. Sports Med..

[35] Ribeiro VB (2020). Effects of continuous and intermittent aerobic physical training on hormonal and metabolic profile, and body composition in women with polycystic ovary syndrome: A randomized controlled trial. Clin. Endocrinol..

[36] Almenning I (2015). Effects of high intensity interval training and strength training on metabolic, cardiovascular and hormonal outcomes in women with polycystic ovary syndrome: A pilot study. PLoS ONE.

[37] Patten, R. K.* et al.* High-intensity training elicits greater improvements in cardio-metabolic and reproductive outcomes than moderate-intensity training in women with polycystic ovary syndrome: A randomized clinical trial. *Hum. Reprod.* (2022). 10.1093/humrep/deac047 35325125

[38] Gomes-Neto M (2017). High-intensity interval training versus moderate-intensity continuous training on exercise capacity and quality of life in patients with coronary artery disease: A systematic review and meta-analysis. Eur. J. Prev. Cardiol..

[39] Martland R (2022). Can high-intensity interval training improve mental health outcomes in the general population and those with physical illnesses? A systematic review and meta-analysis. Br. J. Sports Med..

[40] World Health Organization. *WHO Guidelines on Physical Activity and Sedentary Behaviour: Web Annex: Evidence Profiles*. (2020). 33369898

[41] Brown TA, Chorpita BF, Korotitsch W, Barlow DH (1997). Psychometric properties of the Depression Anxiety Stress Scales (DASS) in clinical samples. Behav. Res. Ther..

[42] Coffey S, Bano G, Mason HD (2006). Health-related quality of life in women with polycystic ovary syndrome: A comparison with the general population using the Polycystic Ovary Syndrome Questionnaire (PCOSQ) and the Short Form-36 (SF-36). Gynecol. Endocrinol..

[43] Hays RD, Morales LS (2001). The RAND-36 measure of health-related quality of life. Ann. Med..

[44] Cronin L (1998). Development of a health-related quality-of-life questionnaire (PCOSQ) for women with polycystic ovary syndrome (PCOS). J. Clin. Endocrinol. Metab..

[45] Jones GL (2004). The polycystic ovary syndrome health-related quality of life questionnaire (PCOSQ): A validation. Hum. Reprod..

[46] Hiam D (2019). The effectiveness of high intensity intermittent training on metabolic, reproductive and mental health in women with polycystic ovary syndrome: Study protocol for the iHIT-randomised controlled trial. Trials.

[47] Bates, D., Mächler, M., Bolker, B. & Walker, S. Fitting linear mixed-effects models using lme4. *arXiv preprint *arXiv:1406.5823 (2014).

[48] Kuznetsova, A., Brockhoff, P. B. & Christensen, R. H. B. lmerTest Package: Tests in Linear Mixed Effects Models. *2017* **82**, 26 (2017).

[49] Wickham H (2019). Welcome to the Tidyverse. J. Open Sour. Softw..

[50] Chekroud SR (2018). Association between physical exercise and mental health in 1·2 million individuals in the USA between 2011 and 2015: A cross-sectional study. Lancet Psychiatry.

[51] Kogure GS (2020). The effects of aerobic physical exercises on body image among women with polycystic ovary syndrome. J. Affect. Disord..

[52] Thomson RL (2010). Lifestyle management improves quality of life and depression in overweight and obese women with polycystic ovary syndrome. Fertil. Steril..

[53] Dokras A (2016). Weight loss and lowering androgens predict improvements in health-related quality of life in women with PCOS. J. Clin. Endocrinol. Metab..

[54] Cooney LG (2018). Cognitive-behavioral therapy improves weight loss and quality of life in women with polycystic ovary syndrome: A pilot randomized clinical trial. Fertil. Steril..

[55] Cabral DF (2019). Exercise for brain health: An investigation into the underlying mechanisms guided by dose. Neurotherapeutics.

[56] Callaghan P, Khalil E, Morres I, Carter T (2011). Pragmatic randomised controlled trial of preferred intensity exercise in women living with depression. BMC Public Health.

[57] Patten RK (2023). Longitudinal affective response to high-intensity interval training and moderate-intensity continuous training in overweight women with polycystic ovary syndrome: A randomised trial. Psychol. Sport Exerc..

[58] Gerber M, Minghetti A, Beck J, Zahner L, Donath L (2019). Is improved fitness following a 12-week exercise program associated with decreased symptom severity, better wellbeing, and fewer sleep complaints in patients with major depressive disorders? A secondary analysis of a randomized controlled trial. J. Psychiatr. Res..

[59] Dixon JB, Dixon ME, O'Brien PE (2003). Depression in association with severe obesity: Changes with weight loss. Arch. Intern. Med..

[60] Greenwood EA (2018). Insulin resistance is associated with depression risk in polycystic ovary syndrome. Fertil. Steril..

[61] Teede HJ (2011). Assessment and management of polycystic ovary syndrome: Summary of an evidence-based guideline. Med. J. Aust..

[62] Deeks AA, Gibson-Helm ME, Teede HJ (2010). Anxiety and depression in polycystic ovary syndrome: A comprehensive investigation. Fertil. Steril..

[63] De Frene V (2015). Quality of life and body mass index in overweight adult women with polycystic ovary syndrome during a lifestyle modification program. J. Obstetr. Gynecol. Neonatal Nurs. JOGNN.

[64] Ladson G (2011). The effects of metformin with lifestyle therapy in polycystic ovary syndrome: A randomized double-blind study. Fertil. Steril..

[65] Arentz S (2017). Combined lifestyle and herbal medicine in overweight women with polycystic ovary syndrome (PCOS): A randomized controlled trial. Phytother. Res. PTR.

