# Supplementary material for: Efficacy of high-intensity interval training for improving mental health and health-related quality of life in women with polycystic ovary syndrome
Source: Sci Rep. 2023 Feb 21;13:3025. doi: 10.1038/s41598-023-29503-1 (PMC9944288; doi:10.1038/s41598-023-29503-1)
Supplement: Supplementary file 1 — Supplementary Information. [file 41598_2023_29503_MOESM1_ESM.docx]

**Efficacy of high-intensity interval training for improving mental health and health-related quality of life in women with polycystic ovary syndrome.**

Patten, R. K., McIlvenna, L., Moreno-Asso, A., Hiam, D., Stepto, N., Rosenbaum, S. and Parker, A. G.

**Supplemental material**


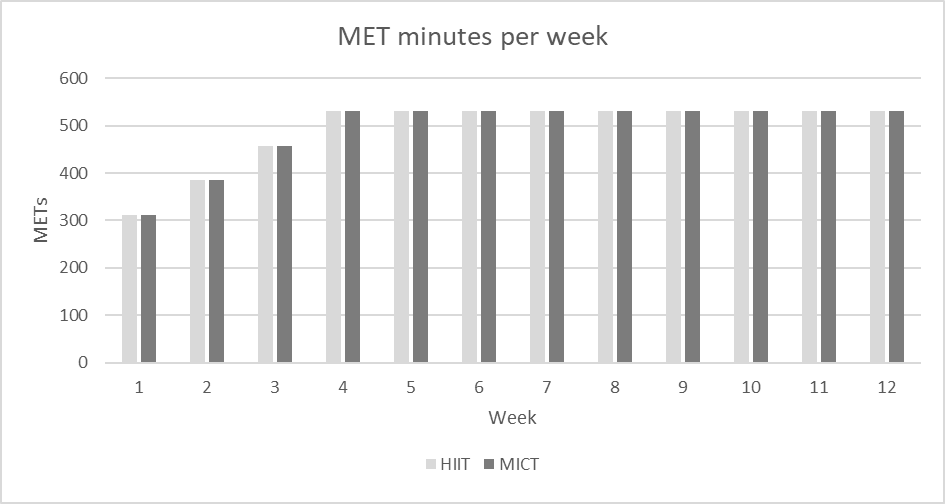
Supplementary Figure 1. Progression of energy expenditure in MET minutes per week for the HIIT and MICT groups.

MET: metabolic equivalent of task, HIIT: high-intensity interval training, MICT: moderate-intensity continuous training.

Supplementary Table 1. PCOS phenotypes at baseline

| PCOS Phenotype | High-intensity interval training N | Moderate-intensity continuous training N |
| --- | --- | --- |
| A (HA, OA, PCOM) | 6 | 6 |
| B (HA, OA) | 3 | 1 |
| C (HA, PCOM) | 1 | 2 |
| D (OA, PCOM) | 5 | 5 |

HA: hyperandrogenism, OA: oligo/anovulation, PCOM: polycystic ovary morphology
